# Traces of water catalyze zinc soap crystallization in solvent-exposed oil paints†

**DOI:** 10.1039/d2cp04861b

**Published:** 2023-01-23

**Authors:** Joen Hermans, Kate Helwig, Sander Woutersen, Katrien Keune

**Affiliations:** a Van‘t Hoff Institute for Molecular Sciences, University of Amsterdam Amsterdam The Netherlands j.j.hermans@uva.nl +31 (0)20 525 6484; b Conservation & Science, Rijksmuseum Amsterdam The Netherlands; c Canadian Conservation Institute Ottawa Canada

## Abstract

The crystallization of metal soaps in polymer matrices is a complex process that affects the stability of oil paintings, as well as the properties of commercial ionomer materials. In the context of conservation of paintings, it is crucial to investigate the influence of solvent exposure on such detrimental chemical processes. Using Fourier transform infrared spectroscopy and a polymer model system that contains metastable amorphous zinc soaps, it is shown that water induces zinc soap crystallization, while solvent swelling alone has no effect. In particular fast-diffusing polar organic solvents with water impurities are able to induce extensive crystallization, delivering high concentrations of water quickly deep into paint layers. Finally, it is demonstrated, both with the model system and real oil paint samples, that even with very short solvent exposure times, significant quantities of crystalline zinc soaps are formed. This strong effect of water impurities in common solvents gives reason to be cautious when conservation treatments are being considered for oil paints that contain zinc white or other water-sensitive chemicals.

## Abstract 2

Water triggers crystallization of zinc soaps in polymer networks, especially when water is mixed with polar organic solvents. This finding has a big impact on conservation of oil paintings.

## Graphical abstract

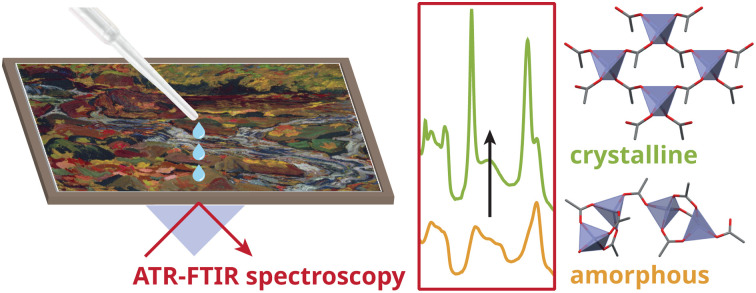

## 1 Introduction

Chemistry plays a central role in paintings’ conservation. In a typical historic oil painting, multiple inorganic pigments and/or organic dyes are bound in a polymerized vegetable oil matrix, interacting with each other and with their environment on a timescale of centuries. Some of these chemical processes can lead to mechanical instability of paint layers or changes in a painting's appearance. To preserve paintings for future generations, a detailed understanding of the chemical processes behind the change and degradation of these complex heterogeneous polymer systems is the key.

One important conservation question concerns the possibility of detrimental effects of solvent exposure during conservation treatments, like varnish removal, surface cleaning, paint consolidation and lining.^1^ In a fully dried paint, most chemical reactions in the polymer network tend to be diffusion-limited and, as a consequence, rather slow. This situation can lead to the establishment of local environments with varying molecular composition and physical conditions near, for instance, pigment interfaces or the paint surface. When solvents diffuse into paint layers, they usually cause considerable polymer swelling,^2,3^ which has the potential to solubilize and transport reactive molecules,^4–6^ trigger undesirable reactions, and cause a rapid change in the properties of paint layers.

In this paper, we focus on the crystallization of zinc soaps as a striking example of chemical reactivity in oil polymer networks. This crystallization process has been linked to several types of paint instability in paintings from the mid-19th century or later when zinc white (ZnO) became available as a common white pigment in artists' oil paints.^7^ In extreme cases, zinc soap formation can lead to delamination and fracture of paint layers or the appearance of globular protrusions on the paint surface.^8–13^ These zinc soaps are insoluble crystalline complexes of zinc ions and saturated fatty acids (FAs, usually palmitic and stearic acid) formed after partial hydrolysis of ester bonds in the triglyceride oil binder and dissolution of ZnO in the oil by reaction with carboxylic acid groups of the oxidized oil.^5,14^ Recent research elucidated the structural evolution of zinc carboxylate species in oil paint systems from ZnO through ionomeric zinc carboxylates to crystalline zinc soaps.^15–17^ This research suggested that the key step leading to a change in paint properties, and therefore, the step one would need to control to prevent problematic changes in paint layers, is the final transition from amorphous to crystalline zinc soaps. A similar link between zinc soap crystallization behaviour and material properties exists in the context of commercial ionomer research, where zinc soaps have been applied to modify the properties of sulfonated poly(ethylene-*co*-propylene-*co*-diene monomer) (SEPDM),^18^ sulfonated polystyrene (SPS),^19^ poly (ethylene-*co*-methacrylic acid) (PEMA)^20^ and carboxylated nitrile butadiene rubber (XNBR).^21^

With its high time resolution and chemical specificity, time-dependent attenuated total reflection Fourier transform infrared (ATR-FTIR) spectroscopy is an ideal technique to monitor the crystallization of metal soaps and the diffusion of solvents in model oil paint polymers.^3,22^ A major finding in previous research was that polymer matrix properties have a large influence on crystallization kinetics and the crystalline polymorphs that form.^22,23^ For conservation of paintings, it is crucial to understand to what extent the absorption of solvents in the paint matrix affects the local kinetics of metal soap crystallization. In order to study these solvent effects, a model system is required that has an oil polymer network structure comparable to aged oil paint, and which contains a population of non-crystalline zinc soaps at room temperature. Given that zinc soaps are generally insoluble in oil polymers under ambient conditions (which is more than 70 °C below their melting temperature),^24^ creating a system that contains such metastable amorphous zinc soaps is rather challenging.

**Fig. 1:**
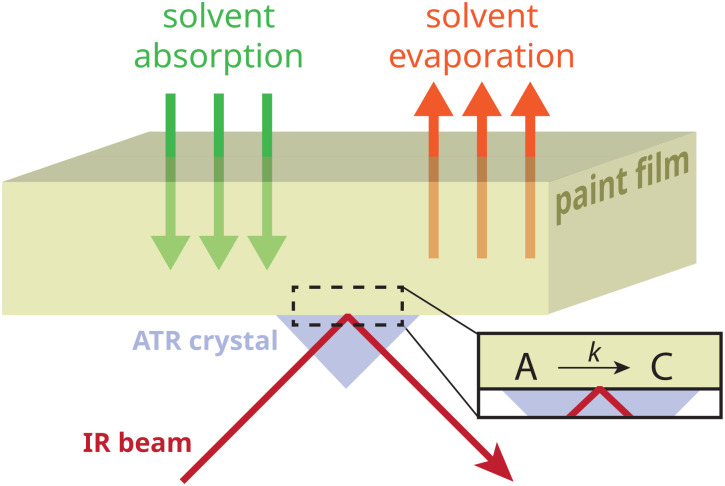
Illustration of the experimental system that is central in this study. With ATR-FTIR spectroscopy, the kinetics of the amorphous (A) to crystalline (C) transition of zinc soaps can be measured (after flipping the film) at an original top surface of a model paint film during solvent absorption and/or evaporation.

Here, we report an aged oil binder model system that contains metastable amorphous zinc soaps generated by heat treatment. To follow the crystallization process of zinc soaps (ZnFA), we employ ATR-FTIR spectroscopy (illustrated in Fig. 1), comparing the effects of various solvents, water impurities, and the duration of solvent exposure. Finally, we investigate whether real aged oil paint indeed contains a population of these metastable amorphous zinc soaps that can be induced to crystallize by short-term solvent exposure.

The reported experiments highlight the complex chemical interplay between pigments, binders and solvents in a typical oil paint system. Moreover, the results provide crucial information on the effects of solvents on reactive oil paint layers, which provides a foundation for conservators when developing treatments for paintings that contain such paint layers.

## 2 Results and discussion

### 2.1 Model system properties

**Fig. 2:**
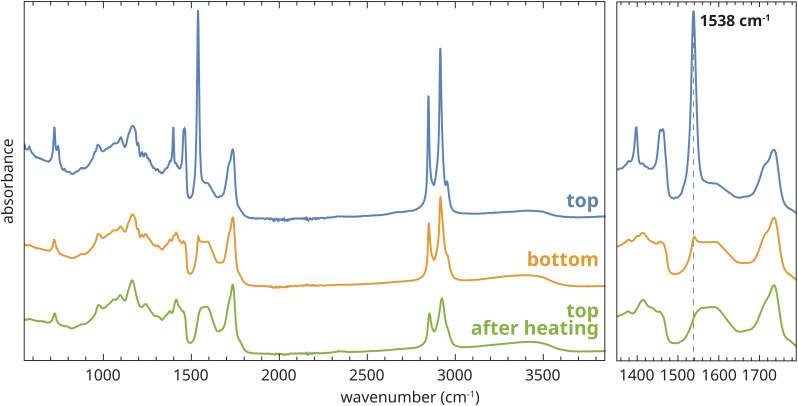
ATR-FTIR spectra collected on the top and bottom side of an aged zinc ionomer film based on linseed oil, and at the top side after heat treatment at 150 °C.

As a model system for an aged oil paint binder, we selected an unpigmented zinc ionomer film (190 μm thick) prepared by co-polymerization of linseed oil with zinc sorbate (2,4-dihexadienoic acid).^25^ Experimental details can be found in Section A.1 of the ESI.† This film was aged for 4.5 years under ambient lab conditions on a glass support, after which it was very brittle but otherwise intact and transparent. ATR-FTIR spectra collected on the top of the film after ageing indicated the presence of a high concentration of type B crystalline ZnFA,^16^ the most clear signatures being the sharp asymmetric carboxylate stretch vibration band at 1538 cm^−1^, the symmetric carboxylate stretch vibration band at 1398 cm^−1^, and the relatively intense CH_2_ stretch vibration bands at 2847 and 2915 cm^−1^ caused by the long alkyl chains in the fatty acid (Fig. 2). The broader carboxylate vibration band (maximum at 1592 cm^−1^) that partially overlaps with the sharp band at 1538 cm^−1^ is due to ionomeric zinc carboxylate groups that adopt a chain and oxo complex conformation.^15,17^ The crystalline ZnFA in this film formed as a consequence of spontaneous hydrolysis of triglyceride ester bonds of the oil polymer network, which generated a population of free saturated fatty acids. In turn, these fatty acids started binding to Zn^2+^ ions by exchanging with polymer-bound carboxylate groups, and finally forming separate crystalline ZnFA phases within the polymer matrix.^17^ These processes are exactly the same as those that lead to zinc soap formation in real oil paint on canvas. Interestingly, the bottom side of the polymer film showed only minor crystallization of ZnFA (Fig. 2). The reason for this difference in properties between the top and bottom surfaces of the film is still unknown. In fact, in contrast to our findings, Osmond and co-workers reported preferential crystallization of zinc soaps at the bottom of commercial zinc white oil paint films, though these paints also contained aluminium stearate and they were applied on PET rather than glass.^7^ For the purposes of the current research, all further measurements on our model systems were carried out on flipped films, *i.e.* with the original top surface of the films in contact with the ATR crystal.

To prepare this model system for the study of ZnFA crystallization, the polymer film was briefly heated to 150 °C (see Section A.2 of the ESI† for experimental details). Above 130 °C, the ZnFA melted and adopted a mixture of the chain and oxo complex structures.^22^ Crucially, the ATR-FTIR spectra collected at the top of the film showed no chemical change after cooling back down to 25 °C for at least 22 h, indicating that ZnFA remained in a metastable amorphous state as oxo and chain complexes for very long periods of time (Fig. 2). For comparison, in a similar experiment performed with mixtures of ZnFA and only partially polymerized fresh linseed oil films, we observed crystallization of ZnFA within minutes after reaching room temperature.^22^ This observation shows that the metastable amorphous state of ZnFA only persists in strongly cross-linked polymer films.

The heat-treated zinc ionomer demonstrates that it is indeed possible for ZnFA to adopt a metastable amorphous state in aged oil polymers at room temperature. In this state, they are indistinguishable from the structures formed by ionomeric zinc carboxylates (*i.e.* zinc ions bound to carboxylate groups in the polymer network), as both amorphous ZnFA and ionomeric zinc carboxylates adopt a mixture of chain and oxo coordination structures around the zinc ions. This polymer system is ideal to study the induction of chemical reactivity by solvent exposure because we can expose the heat-treated ionomer films to solvents and monitor the re-crystallization of ZnFA.

### 2.2 Effects of solvent swelling on zinc soap crystallization

To investigate the effects of solvent swelling on the kinetics of ZnFA crystallization, we collected ATR-FTIR spectra of heat-treated aged zinc ionomer films while exposing them at the top surface to a layer of water, ethanol, acetone or dichloromethane (DCM). These four solvents cover a wide range of swelling power in linseed oil polymer films, from approximately 5% swelling for H_2_O to more than 100% for DCM.^3^ Water, ethanol and acetone are all used routinely in the context of painting conservation treatments such as varnish, overpaint or dirt removal.

**Fig. 3:**
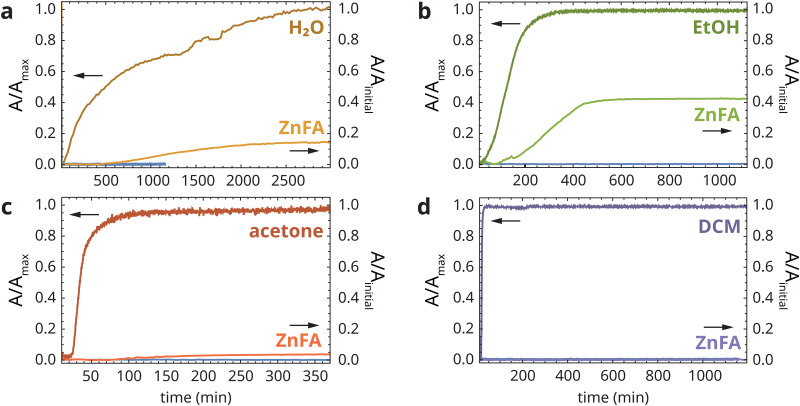
Normalized band areas corresponding to (a) water, (b) ethanol, (c) acetone and (d) DCM, and crystalline ZnFA in heat-treated zinc ionomer films. In each plot, the horizontal blue curve around 0 represents the crystalline ZnFA signal without solvent exposure. Please note the different normalization methods for solvent and ZnFA profiles. While solvent profiles were normalized to their maximum value, ZnFA profiles were normalized to the initial ZnFA band area before heat-treatment.

ATR-FTIR spectra collected continuously during solvent exposure show the appearance of solvent vibration bands, a corresponding decrease in all bands associated with the polymer film due to swelling and, in some cases, the rise of crystalline ZnFA features (see Fig. S1, ESI†). Fig. 3 shows the normalized band area profiles for each of the solvents and crystalline ZnFA (normalization to the initial ZnFA band area prior to heat treatment). Section A.3 of the ESI† describes the data analysis procedure in detail. A comparison of ATR-FTIR spectra prior to heat-treatment, after heat-treatment and after solvent exposure is shown in Fig. S2 (ESI†).

The band area profiles in Fig. 3 clearly indicate that solvent swelling alone does not induce ZnFA crystallization. DCM swelled the polymer film within minutes, but no crystalline ZnFA was detected up to 20 h after the start of exposure (Fig. 3d). In contrast, each of the other three solvents induced some level of ZnFA crystallization soon after the detection of solvent signal in the ATR-FTIR spectra. In all experiments, ZnFA crystallized invariably in the type B polymorph,^16^ which appears to be favourable in solvent-swollen polymer networks. However, the timescale and extent of ZnFA crystallization varied greatly, with ZnFA band areas reaching 4%, 15% and 43% of their maximum values (*i.e.* prior to heat treatment) for acetone, water and ethanol, respectively. This incomplete crystallization of ZnFA is somewhat puzzling, even though it is in line with previous experiments on zinc palmitate crystallization in polymers.^17^ Incomplete ZnFA crystallization has also been observed in sulfonated polystyrene ionomers, where it was attributed to extremely slow crystallization.^19^ In the next section, this result will be discussed in more detail.

In trying to explain these observations of ZnFA crystallization behaviour, it is important to note that no attempt was made to use particularly pure solvents for these experiments, in line with common conservation practices of paintings. As a consequence, the acetone solvent that was used contained traces of water, and the ethanol was in fact an azeotropic mixture containing approximately 4% water. Moreover, it was previously observed that the exchange rate of zinc ions between carboxylate groups was strongly reduced under water-free conditions.^5^ With this information, we can hypothesize that it is in fact the presence of water that triggers the crystallization of ZnFA rather than solvent swelling.

### 2.3 Effects of water on zinc soap crystallization

**Fig. 4:**
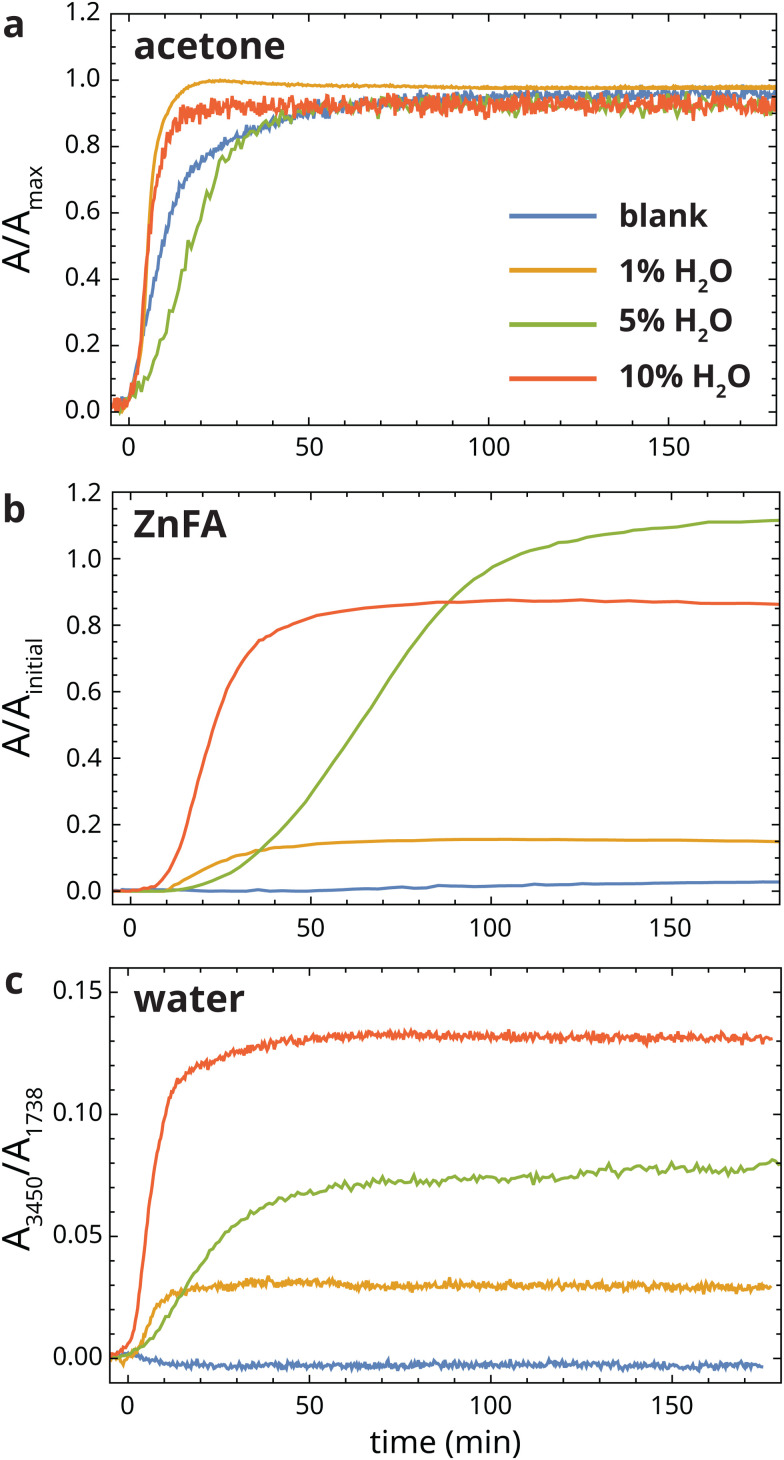
Normalized band areas corresponding to (a) acetone, (b) crystalline ZnFA and (c) water during exposure to heat-treated zinc ionomer films, with 0%, 1%, 5% and 10% (v/v) added to water in the solvent. Please note the different normalization methods for solvent and ZnFA profiles. Acetone profiles were normalized to their maximum value and crystalline ZnFA profiles were normalized to the initial ZnFA band area before heat-treatment. Water profiles reflect OH stretching vibration band intensities (maximum at approximately 3450 cm^−1^) normalized to the ester carbonyl band at 1738 cm^−1^. The onset of the acetone profile was set as *t* = 0 min.

To test the influence of water on ZnFA crystallization, we carried out crystallization experiments in which heat-treated ionomer films were exposed to acetone with increasing concentrations of water. Fig. 4 shows the band area profiles of crystalline ZnFA, acetone and water as a function of time with 0, 1, 5 and 10% (v/v) added water. There was some minor variation in the timescale of acetone diffusion in this set of experiments, probably due to minor differences in film thickness between experiment runs.

Comparing the ZnFA band area profiles in Fig. 4, it is immediately clear that the water concentration in acetone has a strong impact on ZnFA crystallization. Adding just 1% water to acetone already led to extensive crystallization of ZnFA up to 15% of the initial ZnFA band area prior to heat treatment. At 5% and 10% water, the crystalline ZnFA band area reached close to 100% within 100 min after the start of exposure. In all cases, crystallization started when the film was nearly completely swollen with solvent (*i.e.* when the acetone profiles approach a value of 1). In the run corresponding to 5% water, the final zinc soap band area was higher than before the heat treatment of the polymer. The cause for this effect is not clear, but we suspect there could be some diffusion of zinc ions and/or fatty acids into the measurement volume probed by ATR-FTIR in the course of the experiment, leading to minor changes in ZnFA concentrations. Additionally, the effect could be influenced by an overlap between the carbonyl bands of acetone and triglyceride ester, the latter of which is used for normalization prior to data analysis. Fig. S3 (ESI†) shows the comparison of the ATR-FTIR spectra before and after solvent exposure.

There are good reasons to explain the strong influence of water on ZnFA crystallization as a catalytic effect. Considering the changes in zinc carboxylate coordination during crystallization in more detail, the zinc soaps adopt a combination of chain and oxo structures in their amorphous state. These structures can be described as a linear coordination polymer and tetranuclear complex, respectively, where each zinc ion is tetrahedrally coordinated by carboxylate oxygens.^15^ To transform to the 2D coordination polymer structure that characterizes crystalline zinc soaps, where every carboxylate group is bridging between two different zinc ions,^16^ bonds between the Zn^2+^ ions and carboxylate oxygens need to be broken and reformed. As such, it is perhaps more accurate to consider this ZnFA crystallization process as a chemical reaction. This classification is especially true for the transformation from oxo complex (Zn_4_O(FA)_6_) to crystalline zinc soap (Zn(FA)_2_), where additional FA molecules need to be consumed and water is formed.^17^ In the ZnFA crystallization reaction, the transition state will involve an undercoordinated zinc atom and partial charges on both the zinc atom and the carboxylate group. Inside the oil polymer network, we can expect this transition state to be rather unfavourable, leading to extremely slow ZnFA crystallization even when the polymer is swollen with solvents like DCM or water-free acetone that solubilize the fatty acid chains and facilitate molecular motion. However, water can act as a catalyst in the reaction, stabilizing the transition state during crystallization by coordinating to Zn^2+^ ions and shielding charges, leading to faster crystallization.

Beyond the catalytic effect of water, some observations point to additional factors that affect ZnFA crystallization behaviour. The fact that ZnFA crystallization is incomplete at low water concentrations (see also Fig. 3) seems to suggest that water is a reactant in the crystallization process rather than a catalyst. However, the water band area profiles in Fig. 4c clearly remain constant as ZnFA crystallization progresses, which disproves this idea. Moreover, it is difficult to see how water could be consumed in a reaction towards crystalline ZnFA. Instead, we suspect that the water concentration inside the polymer network is not homogeneous at lower water concentrations. Previously, we observed that chain complex zinc carboxylates remain present in oil polymer networks under high humidity conditions,^17^ even though this complex is only stable under completely water-free conditions in solution.^26^ Additionally, DiTullio and co-workers observed dynamically distinct populations of water in oil paint binders with NMR spectroscopy.^27^ Both these observations could point to an inherent inhomogeneity in water distribution in oil polymer networks. In support of this notion of water inhomogeneity, there are many studies of the spinodal decomposition of solutions of triglycerides and water in acetone, ethanol and other solvents^28–30^ into one phase rich in water and solvent, and a second rich in oil and low in water. In triglyceride polymer networks like those in oil paints, the restricted movement of polymer segments would mean that such a phase separation process, if it still exists, could only occur on the micro- or even nanoscale. While this speculative explanation for ZnFA crystallization behaviour remains to be investigated in depth, water inhomogeneity could cause a situation where only a fraction of amorphous ZnFA is reached by water at low water concentrations, leading to incomplete crystallization.

The role of the organic solvents in this ZnFA crystallization process is especially important in the context of conservation of paintings. Exposure to pure water induced only limited ZnFA crystallization on the timescale of a typical conservation treatment (Fig. 3a), because the diffusion of water in unswollen oil polymer films is exceptionally slow and the maximum water concentration is low, even at equilibrium swelling.^3^ As such, short-term exposure to water is likely to only affect the surface region of a paint layer and have only a limited effect on the paint interior. However, polar organic solvents like ethanol and acetone that are characterized by fast diffusion and high swelling power^3^ allow water impurities in these solvents to diffuse orders of magnitude faster and reach high concentrations deep inside the polymer network. As such, these solvents effectively act as an efficient delivery system of water deep into paint films.

### 2.4 Short-timescale solvent exposure

So far, we have considered experiments in which heat-treated zinc ionomer films were completely submerged in liquid solvent for long periods of time. To make the connection to conservation practice of paintings, we carried out short-term solvent exposure experiments that mimic more closely the application of solvent to a painted artwork during a typical conservation treatment. After heat treatment of the polymer films, droplets of solvent were applied to the sample one by one with a syringe, applying each drop just before the previous drop had evaporated completely. As soon as any solvent signal was detected at the bottom of the film in the ATR-FTIR spectra, solvent application was immediately stopped and the solvent was left to evaporate. In practice, 3 or 4 solvent droplets were sufficient in this procedure.

**Fig. 5:**
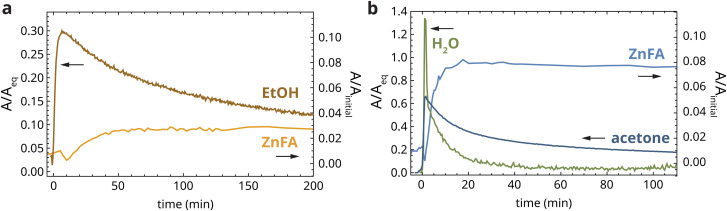
Normalized band area profiles for solvents and crystalline ZnFA during short-timescale exposure to (a) ethanol (96%) and (b) 5% water/acetone (v/v). Please note the different normalization methods for solvent and ZnFA profiles. Crystalline ZnFA profiles were normalized to the initial ZnFA band area before heat-treatment, while ethanol, acetone and water profiles were normalized to the maximum solvent band area at equilibrium swelling (*A*_eq_), and are relative to the ester carbonyl vibration band at 1738 cm^−1^. The onset of the solvent profile was set as *t* = 0 min.

Fig. 5a shows the band area profiles of ethanol and crystalline ZnFA during the dropwise application of 96% ethanol to a heat-treated zinc ionomer film. During this application, 30% of the equilibrium swelling band area (*A*_eq_) was reached at a depth of 190 μm after approximately 7 min, after which the ethanol concentration started decreasing slowly. Surprisingly, ethanol signal could still be detected after 19 h at 3% of *A*_eq_. This observation demonstrates that solvent desorption is a much slower process than absorption, and that solvent remains present beneath the surface of paint films long after application has finished and the surface appears dry. A mismatch between absorption and desorption times in oil paint films has been observed before,^31^ but in our experiment, the effect is much stronger. The slow desorption process is at least partially due to the fact that the solvent diffusion coefficient increases strongly with the degree of swelling.^3^ As such, during absorption solvent quickly diffuses further into the film through already swollen regions of the polymer network. In contrast, during desorption the region of the film close to the film surface has a low solvent concentration and diffusion coefficient, effectively acting as a barrier. Importantly, in the period of relatively high ethanol and water concentration at the bottom of the film (20–60 min), there was a small but clearly measurable increase in the band area corresponding to crystalline ZnFA, up to approximately 3% of the initial band area (see also Fig. S4, ESI†). While this increase is not large, we can expect repeated solvent exposure to paintings to have a significant accelerating effect on crystalline ZnFA formation.

In the case of the application of 5% (v/v) water/acetone, the solvent and crystalline ZnFA profiles paint an even more detailed story of the chemical changes inside the polymer network during solvent exposure. Fig. 5b shows that the acetone band area rises to 65% of *A*_eq_ during dropwise application. Crucially, on this timescale of several minutes, the band area of water relative to the oil polymer reached up to 135% of the band area during swelling in pure water. This water diffusion timescale is faster by approximately three orders of magnitude than during exposure to pure water (Fig. 3a). This important result highlights the potential risks of exposure of paint films to polar solvents like acetone that contain water impurities, even when exposure times are short. Traces of acetone could be detected up to approximately 14 h after stopping droplet application. In contrast, the water signal decreased much more rapidly, reaching pre-application levels after approximately 40 min. The reason for the mismatch in desorption timescale for acetone and water in this experiment is not yet clear, although it could be related to the phase separation phenomena discussed in the previous section.

Despite the relatively brief presence of high water concentrations in the polymer network, we observed clear signs of crystallization of ZnFA, up to 8% of the initial band area before heat treatment (see also Fig. S4, ESI†). However, as the water band area decreased below 20%, crystallization halted. Once again, these results demonstrate the direct link between ZnFA crystallization and water concentration in oil polymer networks.

### 2.5 Realistic paint systems

The experiments in the previous sections have demonstrated that, if an oil polymer contains a sizable concentration of amorphous ZnFA, its crystallization will be induced by water present in polar solvents. To complete this study and link to conservation practice of paintings, it is crucial to investigate whether real aged zinc white paints indeed contain a metastable population of amorphous zinc soaps that can be induced to crystallize by short-term solvent exposure.

**Fig. 6:**
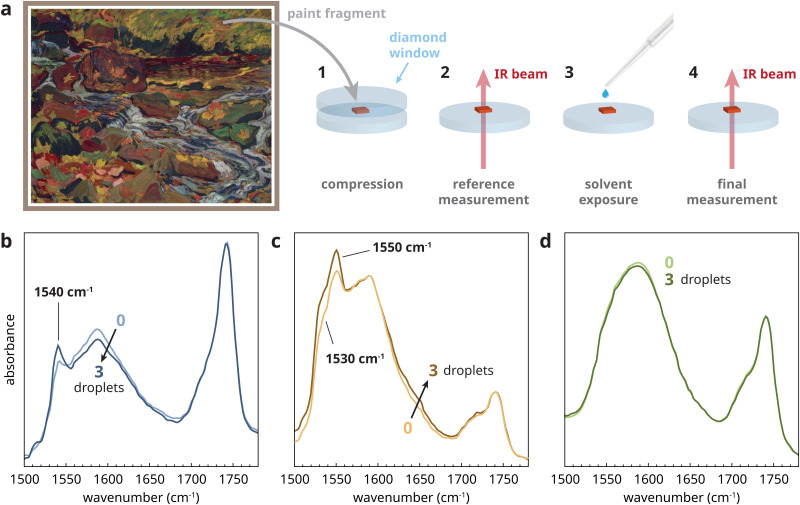
(a) *Leaves in the Brook* by J. E. H. MacDonald (1873–1931) (made *circa* 1918, oil on board, 21.3 × 26.6 cm, McMichael Canadian Art Collection, Kleinburg, gift of the Founders, Robert and Signe McMichael, 1966.16.35), and a schematic of the experimental procedure for tests on real paint samples. (b–d) Transmission FTIR microscopy spectra showing the effect of exposure to 3 small drops of 10% (v/v) water in ethanol for three different real paint samples. (b) A Grumbacher commercial zinc white/titanium white paint tube from the 1970s, painted out on the glass and aged for 10 years. (c) A sample of pink paint (zinc white and alizarin red) taken from the painting by J. E. H. MacDonald. (d) A sample of light blue paint (zinc white, ultramarine, viridian) taken from the same painting.

A sample was selected from a paint film made from a commercial Grumbacher zinc white/titanium white paint tube dating from 1970 or earlier that was found in the studio of Canadian artist J. B. Taylor. The paint was applied to a glass slide, and dried and aged for 10 years under ambient conditions. A small sample, compressed in a diamond cell to several micrometers thick, was exposed to three small droplets of 10% (v/v) water in ethanol and allowed to evaporate in between each droplet (see Fig. 6a and Section A.3 of the ESI† for experimental details). Fig. 6b shows the transmission FTIR spectra before and after exposure, normalized to the triacylglyceride ester carbonyl stretch vibration at 1740 cm^−1^. Prior to solvent exposure, a main broad non-crystalline zinc carboxylate band was observed in this sample, with a sharp band at 1540 cm^−1^ indicating the presence of type B crystalline ZnFA. After exposure to the solvent droplets, the type B crystalline ZnFA band showed a small but clear increase, while the amorphous zinc carboxylate band decreased. Very similar results were found with 10% (v/v) water in acetone mixture and with pure water, while pure acetone caused no measurable change (see Fig. S5, ESI†).

To expand the scope of these tests, we also considered two oil paint samples, each containing zinc white, from the painting *Leaves in the Brook* painted *circa* 1918 by Canadian Group of Seven artist J. E. H. MacDonald (Fig. 6a). Fig. 6c shows that a sample of pink paint containing zinc white and alizarin red had a rather high concentration of non-crystalline zinc carboxylates relative to the ester band, and a set of bands at 1530 and 1550 cm^−1^ indicative of type A crystalline ZnFA.^16^ Following the same exposure procedure as before, the crystalline ZnFA bands clearly increased with exposure to water in ethanol, again with similar results after water in acetone and pure water exposure. In contrast, a sample from a light blue passage of paint from the same painting (Fig. 6d) showed only amorphous zinc carboxylates prior to solvent treatment and no measurable change after solvent exposure. This result means either that the sample only contained ionomeric zinc carboxylates and no zinc soaps, or that zinc soaps were present but could not be induced to crystallize under these conditions. In either case, the experiment suggests that this particular light-blue paint is less prone to the solvent-induced chemical change.

These experiments on real paint samples demonstrate the large chemical variety that is the reality of studying historical oil paint materials. Three different aged oil paints containing zinc white, two of which are from the same painting, each behave rather differently. However, the key result is that the zinc white oil paints that are part of art objects really do contain a population of amorphous ZnFA in some cases, and that exposure to water, even on very short timescales, induces partial crystallization of these soaps. The strong crystallization effect of pure water exposure in these experiments is most likely due to the very thin nature of the samples, which eliminates the influence of water transport kinetics on the rate of ZnFA crystallization. It is not yet clear whether it is the presence of crystalline ZnFA prior to solvent exposure or other factors that determine whether water exposure is a considerable risk for a given zinc white paint. However, this current method of exposing a very small paint sample (approximately 50 μm in diameter) to water or solvents with water impurities on a diamond window and monitoring chemical change with transmission FTIR microscopy could be suitable as a risk assessment tool prior to conservation treatments.

## 3 Conclusions

The experiments based on FTIR spectroscopy described in this study provide important insights into water-induced chemical changes in oil paint polymers. It is now clear that zinc soaps can persist in an amorphous state in aged oil polymers at room temperature, despite being well below their melting point. While the crystallization of these amorphous soaps is not affected by solvent swelling alone, the presence of water does induce crystallization, probably by stabilizing charged groups during the change in zinc carboxylate coordination that accompanies crystallization. Therefore, polar organic solvents with high diffusion coefficients like acetone and ethanol can play an important role in this crystallization process by facilitating the transport of relatively high concentrations of water deep into oil paint layers.

Moving closer to realistic conservation treatments, we showed that even exposure to only a few solvent droplets that contain 4–5% water can induce the formation of measurable quantities of crystalline zinc soaps. The strength of this accelerating effect is aided by the striking observation that solvent desorption is extremely slow, leading to solvent desorption lasting many hours even with exposure times of just a few minutes. Finally, we demonstrated an experimental method based on transmission FTIR microscopy to measure the chemical response to solvent exposure of minute real paint samples from artworks. Tests on three samples showed that solvent-induced zinc soap crystallization can also occur in real historic oil paint, though not all oil paints that contain zinc white reacted in the same way.

This work on the molecular response of oil paint to solvent exposure suggests that special caution is warranted when solvent-based conservation treatments are being considered for oil paint that contains zinc white. Furthermore, we suspect that commercial ionomer materials that contain zinc soaps as part of their formulation could be similarly sensitive to water and/or solvent exposure.

## Author ontributions

J. H.: conceptualization, formal analysis, investigation, methodology, visualization, writing – original draft, funding acquisition. K. H.: investigation, resources, writing – review & editing. S. W.: writing – review & editing. K. K.: writing – review & editing.

## Conflicts of interest

There are no conflicts to declare.

## Supplementary Material

CP-025-D2CP04861B-s001
