# Supplementary material for: Traces of water catalyze zinc soap crystallization in solvent-exposed oil paints
Source: Phys Chem Chem Phys. 2023 Jan 23;25(7):5701–9. doi: 10.1039/d2cp04861b (PMC9930726; doi:10.1039/d2cp04861b)
Supplement: CP-025-D2CP04861B-s001 [file CP-025-D2CP04861B-s001.pdf]

Electronic Supplementary Material (ESI) for Physical Chemistry Chemical Physics.
This journal is © the Owner Societies 2023

Supporting Information

# Traces of water catalyze zinc soap crystallization in solvent-exposed oil paints

Joen Hermans,* Kate Helwig, Sander Woutersen and Katrien Keune

*Van 't Hoff Institute for Molecular Sciences, University of Amsterdam,*
*PO box 94157, 1090GD Amsterdam, The Netherlands.*
*E-mail: j.j.hermans@uva.nl*

## Contents

**A Methods** **2**

A.1 Materials . . . 2

A.2 ATR-FTIR measurements . . . 2

A.3 Transmission FTIR measurements . . . 3

A.4 Data analysis . . . 3

**B ATR-FTIR spectrum series of heat-treated zinc ionomer film during ethanol exposure** **5**

**C ATR-FTIR spectra, exposure to different solvents** **6**

**D ATR-FTIR spectra, exposure to increasing water concentrations** **7**

**E ATR-FTIR spectra, short-term solvent exposures** **8**

**F Transmission FTIR spectra of real paint samples, short-term solvent exposure** **9**

# A Methods

## A.1 Materials

Zinc ionomer films were prepared by co-polymerization of linseed oil and zinc sorbate. Zinc sorbate was prepared as described previously (Baij, L., Hermans, J. J., Keune, K., & Iedema, P. D. (2018). *Macromolecules*, 51(18), 7134–7144. DOI: 10.1021/acs.macromol.8b00890). Zinc sorbate (155 mg) and cold-pressed untreated linseed oil (Kremer Pigmente, 200 mg) were mixed together using a mortar and pestle to a smooth paste. The mixture was spread onto a glass slide using a draw-down bar, and cured overnight in an air-circulated oven at 150 °C. The resulting dark orange transparent film had a thickness of approximately 190 $\mu$ m. After curing, the film was left to age for 4.5 years under laboratory conditions, after which it was intact but very brittle. For ATR-FTIR measurements, small fragments of the film (approximately 2×3 mm) were cut and lifted carefully off the glass support.

The Grumbacher paint was sourced from the studio of Canadian artist J.B. Taylor (1917-1970). The tube dates from 1970 or earlier, and its label describes the contents as "Superba white, non-yellowing titanium" with "Guaranteed ingredients: pure titanium dioxide and zinc oxide in highly purified finest quality oil". Some of the paint from this tube was painted out on a glass slide in 2012, after which is was dried/aged under ambient conditions for 10 years. The two samples from a historic painting were taken from *Leaves in the Brook* By J.E.H. MacDonald which was painted in ca. 1918. In all three cases, a small sample (approximately 50 $\mu$m in diameter) was cut with a scalpel and investigated with transmission FTIR spectroscopy.

## A.2 ATR-FTIR measurements

ATR-FTIR spectra were collected on a Frontier spectrometer (Perkin Elmer) equipped with a heatable diamond GladiATR module (Pike Technologies). Individual spectra were collected as a single scan and at 4 cm$^{-1}$ resolution. Measurement runs were carried out as follows:

1. a spectral background was collected at 25 °C;

2. a sample of the zinc ionomer film was placed on the centre of the ATR crystal, and automated spectrum collection was started with one spectrum per 30 s. For runs that included long-term solvent exposure, a custom sample cell was placed over the sample to allow submersion of the sample (macromolecules paper). For short-term solvent exposures, the sample cell was not used. Instead, the sample film was pressed onto the ATR crystal using the pressure clamp of the ATR module during heat treatment.

3. the ATR top plate was heated to 150 °C, and kept at this temperature for 3 min;

4. the sample was brought back to 25 °C by stopping the heating module and placing heavy brass ring on the ATR top plate;

5. after reaching 25 °C, the temperature was kept constant. The sample was exposed to a solvent of choice by filling the sample cell chamber with 1-2 mL of solvent using a syringe. For short-term solvent exposure experiments, the pressure clamp was carefully released, and individual droplets

of solvent were applied to the film surface using a syringe. Spectrum collection was continued for up to 50 h, or until no spectral changes could be observed.

## A.3 Transmission FTIR measurements

Transmission FTIR spectra were collected on a Hyperion 2000 microscope (Bruker) with an MCT detector interfaced to a Tensor 27 spectrometer. Samples were measured in transmission mode by co-adding 200 scans and using a 4 $cm^{-1}$ resolution.

The examined paint fragments were on the order of 50 $\mu$m in diameter. Each sample was mounted on a Spectra-Tech low-pressure diamond anvil cell composed of two Type IIA diamonds with a 0.6 mm-diameter free working area. The sample was pressed between the two diamonds to achieve a thickness of a few micrometers and the upper diamond was removed. The open diamond was placed on the stage of the microscope, and an area of the sample was masked using a variable rectangular aperture of approximately 50 $\times$ 100 $\mu$m. After an initial spectrum was collected, the sample was treated with three subsequent drops of solvent, allowing the solvent to evaporate between each application. The diamond cell was then replaced on the microscope stage in the same orientation and without changing the apertures; this ensured that the same area was analyzed. For solvent application, a small drop, hanging from the end of a glass pipette was gently placed on the surface of the diamond to the entire working area.

## A.4 Data analysis

All data was processed and analyzed using custom Wolfram Mathematica scripts. The data analysis method is the nearly the same as described in previous work, where it was extensively tested and optimized (Hermans, J., Zuidgeest, L., Iedema, P., Woutersen, S., & Keune, K. (2021). *Phys Chem Chem Phys*, 23, 22589–22600. DOI: 10.1039/d1cp03479k). The Supporting Information of that publication contains an example Mathematica Notebook file that provides an implementation of all data processing steps.

The following pre-processing steps were executed for each spectrum in a measurement series:

- spectra were normalized to the ester C=O stretch vibration band around 1738 $cm^{-1}$ to account for variations in sample contact between samples or within a series.
- spectra were chopped to the region of the asymmetric carboxylate stretch vibration band (1500–1650 $cm^{-1}$).
- a linear background was subtracted between the edges of the spectral window.

A hybrid non-linear spectral fit was performed with the `NonlinearModelFit` function in Mathematica, where the carboxylate band envelope was modeled as a sum of a pure reference spectrum for type B ZnFA and a set of Gaussian bands for oxo (1 band) and chain complexes (3 bands). An example of such a fit is shown in the figure below. In this set of experiments, no signals corresponding to type A ZnFA were observed, so this zinc soap species was omitted from the fit. Constraints were imposed on the Gaussian band parameters. The position and width of each Gaussian band was allowed to vary

slightly around the typical value for each parameter, and the relative magnitude of the two largest bands of the chain complex spectrum was limited to a small window. All fits converged well, and for each series, at least 10 fits at various points of the measurement run were inspected manually to ensure the absence of fitting artefacts. The resulting band intensity profiles corresponding to crystalline ZnFA were normalized to their intensity prior to heat treatment.

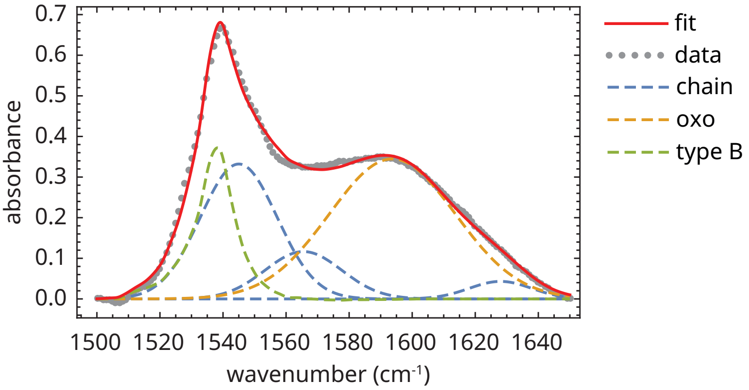


Example of a fit to the carboxylate band envelope with three different zinc carboxylate species. The type B band shape was taken from a pure reference spectrum, while the oxo and chain bands were modeled with Gaussian band shapes.

Solvent concentration profiles were obtained by integration of characteristic solvent bands (529 $cm^{-1}$ for acetone, 731 $cm^{-1}$ for DCM, 1045 $cm^{-1}$ for ethanol) after linear background subtraction. For water, the broad OH band was integrated after subtraction of the spectrum just prior to water exposure to avoid contributions of the $-OH$ groups inherently present in the polymer. A small jump in the signal in the experimental run with pure water, probably due to water being drawn into a small gap between the sample film and the ATR crystal, was corrected by shifting the signal vertically to align with the previous trend.

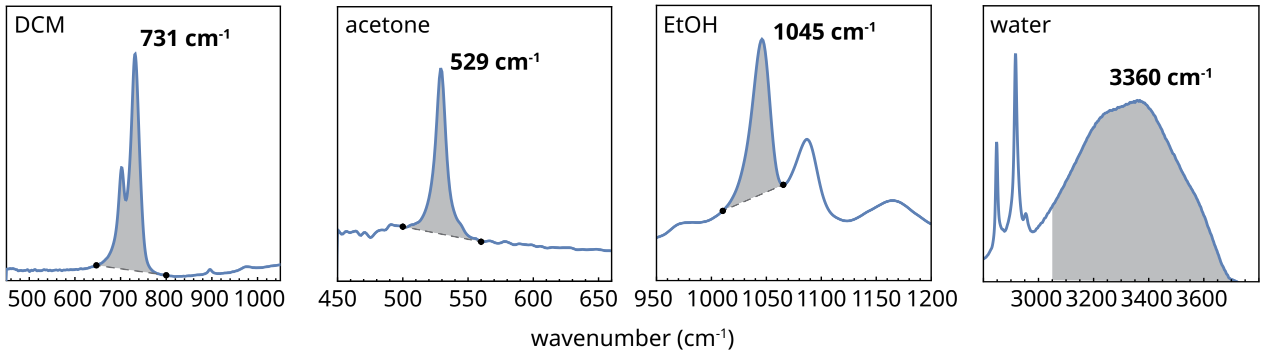


Integration procedure for the four solvents. All spectra are normalized final spectra from solvent exposure runs, except for water, where the first spectrum was subtracted from the final spectrum to obtain a difference spectrum. This difference spectrum contains contributions from $-CH_2$ and $-CH_3$ stretch vibrations, because these bands increase during ZnFA crystallization.

# B ATR-FTIR spectrum series of heat-treated zinc ionomer film during ethanol exposure

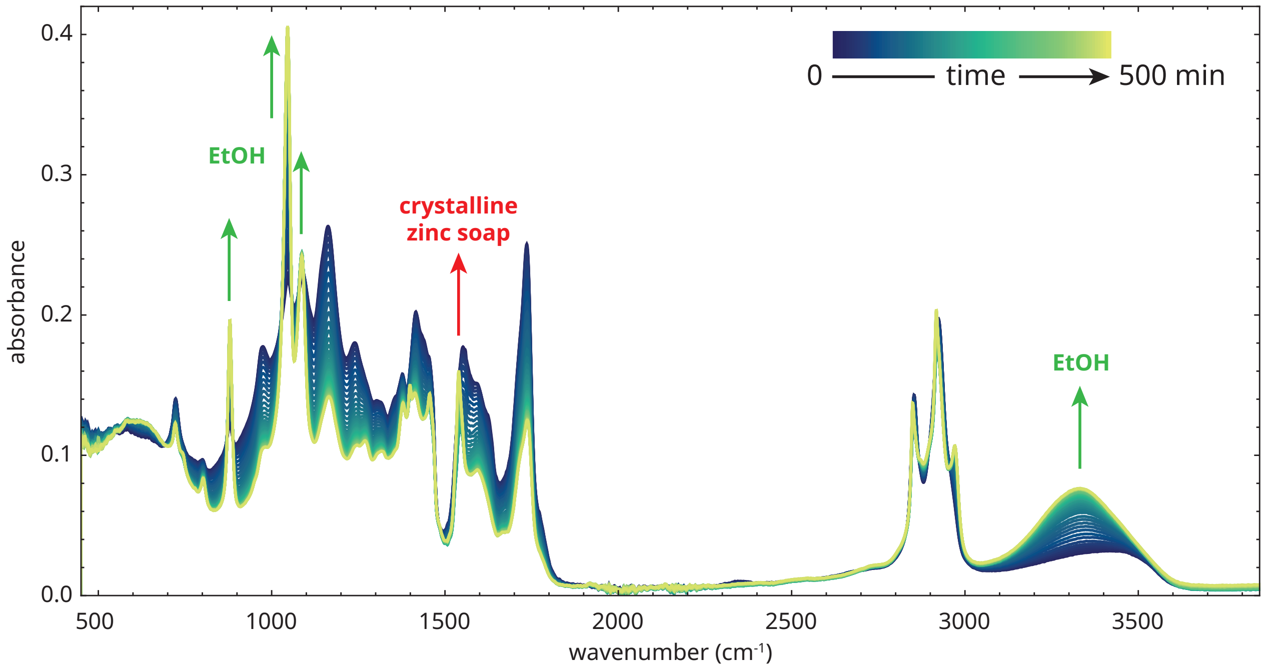


**Figure S1:** Series of ATR-FTIR spectra collected on a heat-treated linseed oil-based zinc ionomer film during 500 min exposure to 96% ethanol. The spectra show a rapid increase in the bands associated with ethanol and simultanseous decrease in all other spectra features due to swelling of the film. On a longer timescale, the bands associated with crystalline zinc soap increase in intensity (in particular the asymmetric COO stretch vibration at 1538 $cm^{-1}$).

## C ATR-FTIR spectra, exposure to different solvents

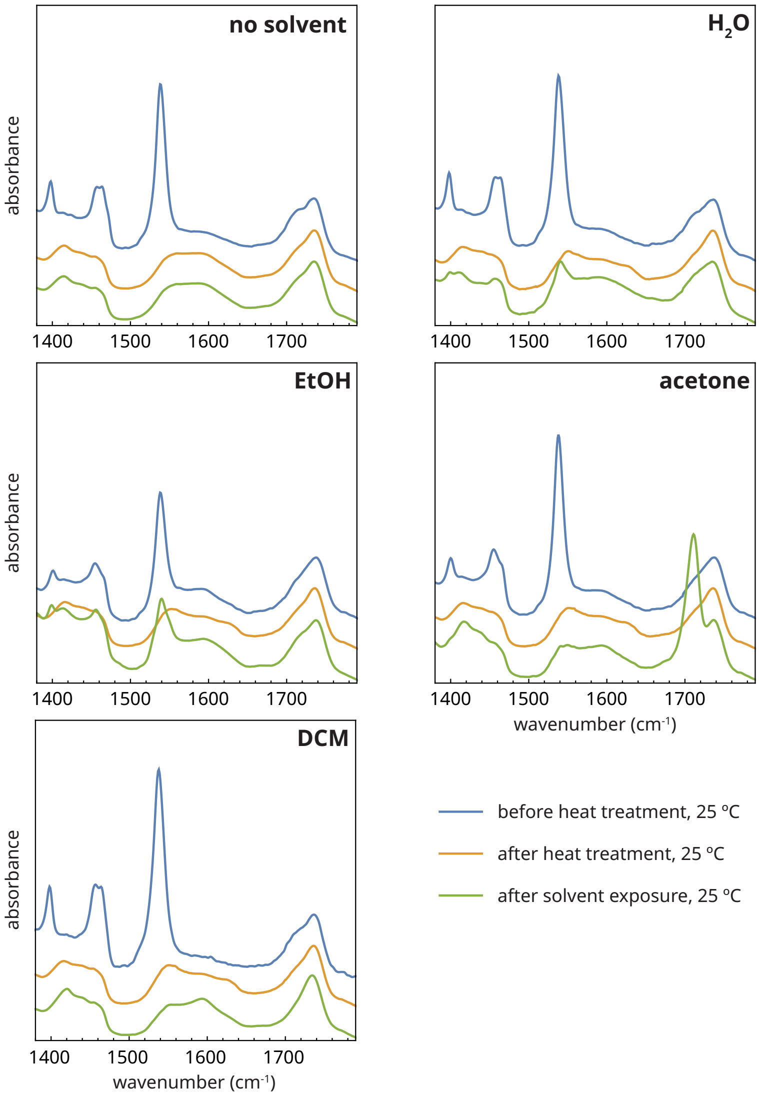


**Figure S2:** Series of ATR-FTIR spectra comparing the C=O region of linseed oil-based zinc ionomer samples before heat treatment, after heat treatment, and after solvent exposure.

## D ATR-FTIR spectra, exposure to increasing water concentrations

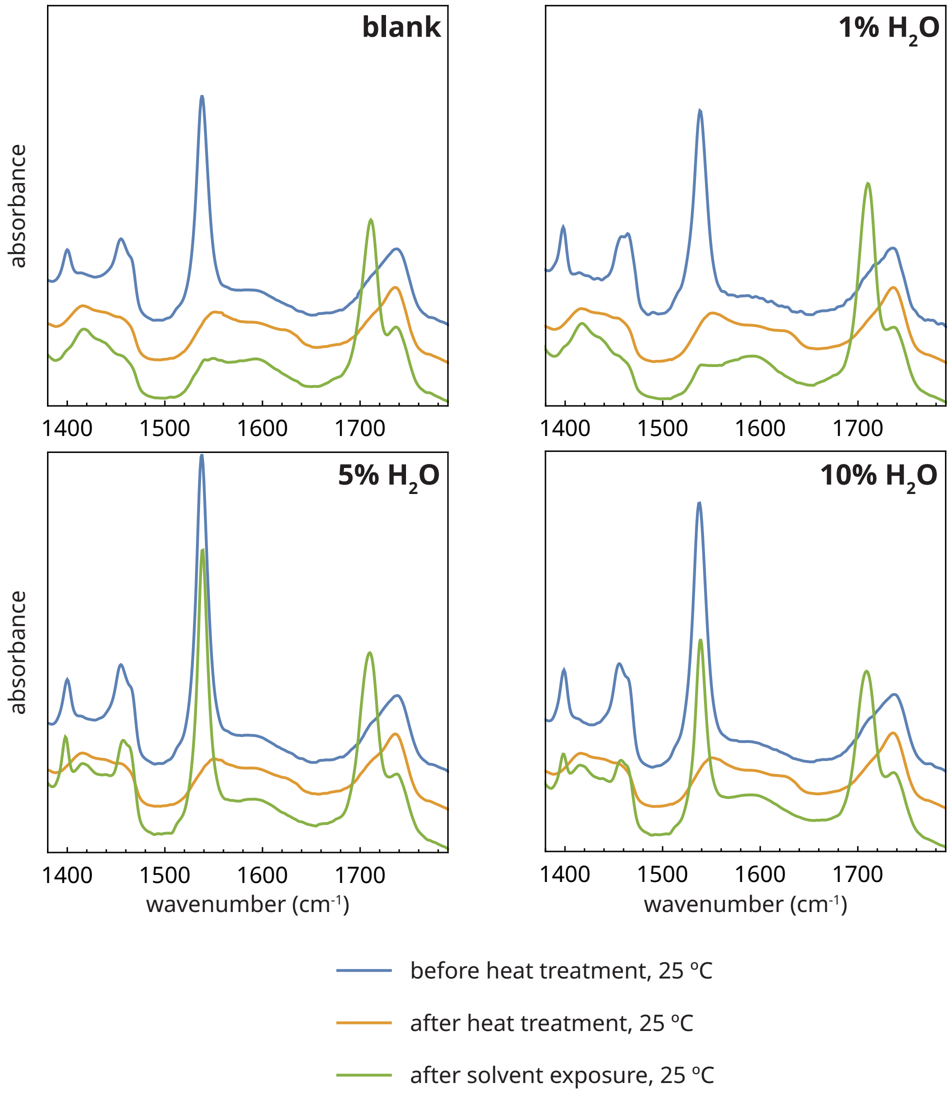


**Figure S3:** Series of ATR-FTIR spectra comparing the C=O region of linseed oil-based zinc ionomer samples before heat treatment, after heat treatment, and after exposure to acetone with increasing concentrations of water.

# E ATR-FTIR spectra, short-term solvent exposures

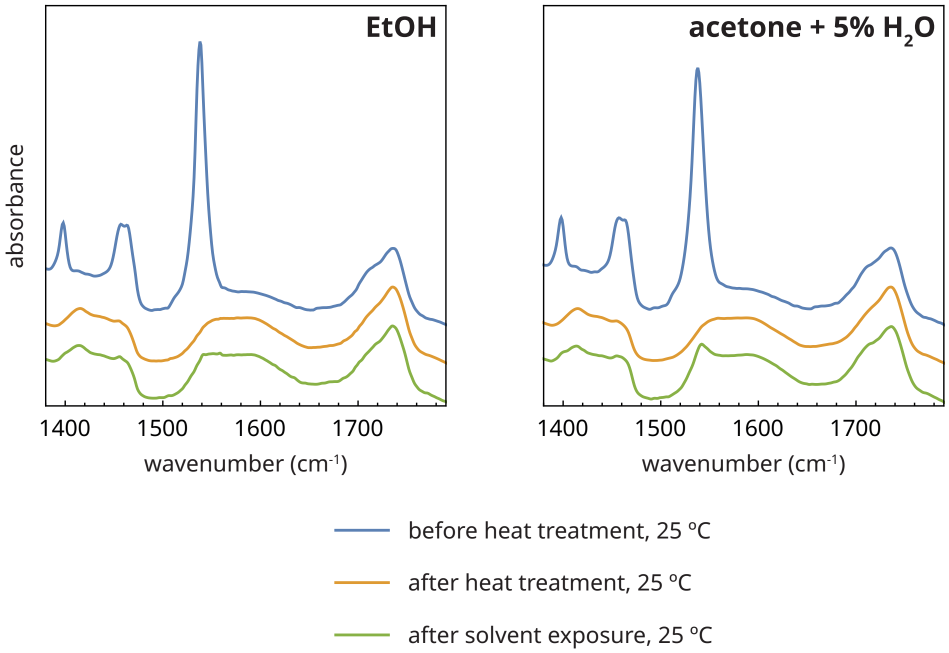


**Figure S4:** Series of ATR-FTIR spectra comparing the C=O region of linseed oil-based zinc ionomer samples before heat treatment, after heat treatment, and after exposure to 96% ethanol or 5% water in acetone.

# F Transmission FTIR spectra of real paint samples, short-term solvent exposure

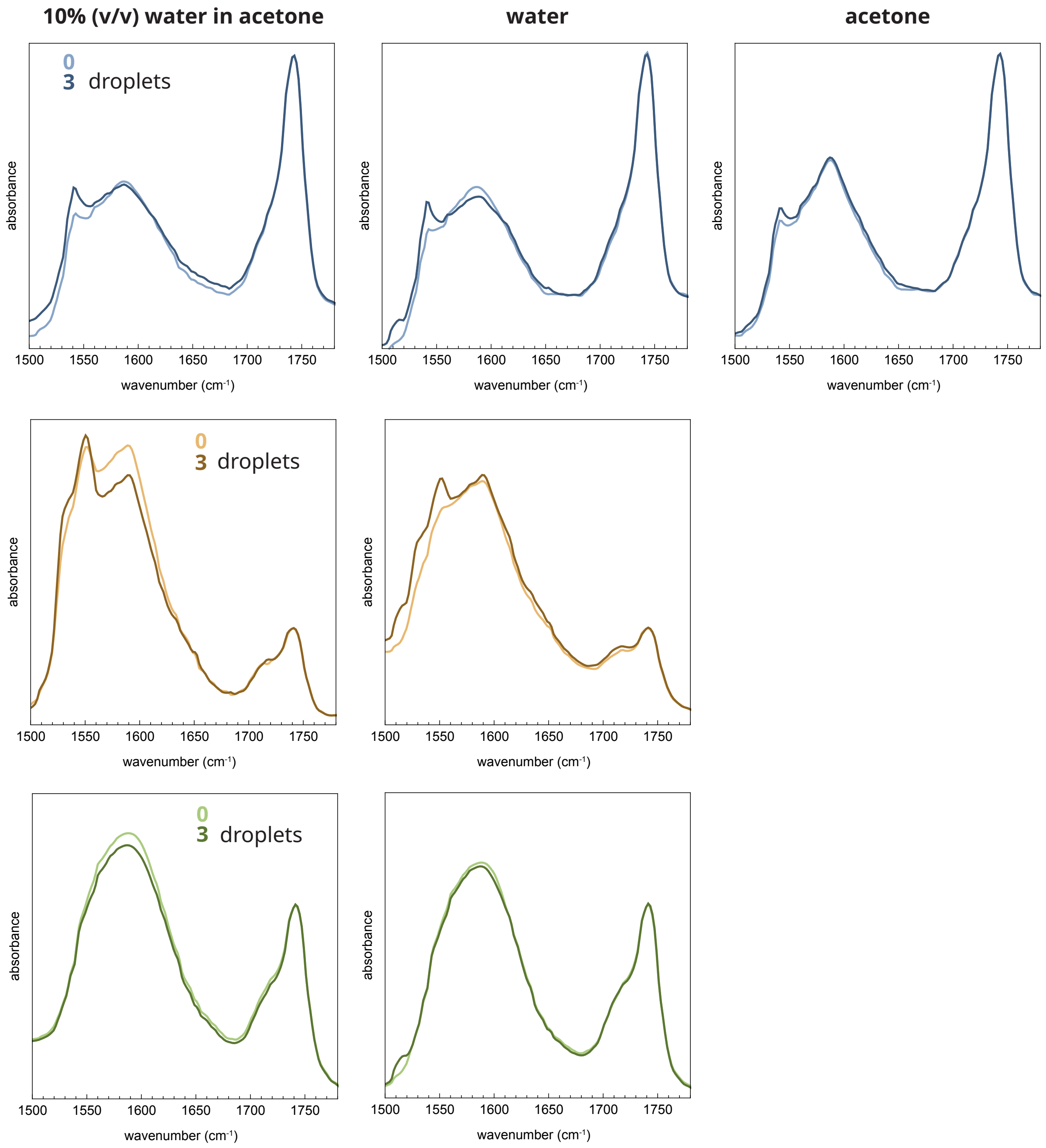


**Figure S5:** Transmission FTIR spectra showing the effects of exposure to up to 3 drops of 10% (v/v) water in acetone, pure water and pure acetone for three different real paint samples. (top row) A Grumbacher commercial zinc white/titanium white paint tube from the 1970s, painted out on canvas and aged for 10 years. (middle row) A sample of pink paint (zinc white and alizarin red) taken from the painting *Leaves in the Brook* by J.E.H. MacDonald (painted circa 1918-1919). (bottom row) A sample of light blue paint (zinc white, ultramarine, viridian) taken from the same painting. Spectra were normalized to the ester carbonyl band at 1740 $cm^{-1}$.
